# Pulmonary Function and Influencing Factor Investigation for Rural Homemakers in the Fenwei Plain, China

**DOI:** 10.3390/toxics13121061

**Published:** 2025-12-07

**Authors:** Rong Feng, Kaiyuan Wang, Hongmei Xu, Yunxuan Gu, Liu Yang, Jian Sun, Zhenxing Shen

**Affiliations:** 1 Department of Environmental Sciences and Engineering, Xi’an Jiaotong University, Xi’an 710049, China; fengrong@stu.xjtu.edu.cn (R.F.); wangkaiyuan@stu.xjtu.edu.cn (K.W.); guyunxuan@stu.xjtu.edu.cn (Y.G.); yangliu2013@xjtu.edu.cn (L.Y.); sunjian0306@mail.xjtu.edu.cn (J.S.); zxshen@xjtu.edu.cn (Z.S.); 2 State Key Laboratory of Loess and Quaternary Geology, Institute of Earth Environment, Chinese Academy of Sciences, Xi’an 710049, China

**Keywords:** pulmonary function, influence factor, rural homemakers, lung function

## Abstract

To understand the pulmonary function and main influencing factors of homemakers in rural Xi’an, a representative city in Northwest China, 72 housewives (61 ± 9 years old) were randomly selected from the rural area of Lantian, Xi’an. The questionnaire survey and pulmonary function test were performed on the subjects in winter and summer, respectively. The general linear model and variance analysis were used to analyze the influencing factors of pulmonary function. Key lung function indices included Vital Capacity (VC: 2.06 ± 0.48 L), Forced Expiratory Volume in First Second (FEV1: 1.91 ± 0.52 L), Forced Vital Capacity (FVC: 2.23 ± 0.59 L), and the FEV1/FVC ratio (0.86 ± 0.07). Several factors were found to cause impairment of pulmonary function. Age has the greatest effect on various indicators of lung function (Eta: 22.3%); the effect of indoor ventilation, season, and second-hand smoke (SHS) exposure on pulmonary function was comparable (3.2–5.9%). There were significant differences on most pulmonary function indices between four age groups (*p* = 0.000–0.005), and the age of <57 years old displayed the highest lung function index values. The lung function of the ventilation group was better than that of the non-ventilation group. And the lung function of the non-SHS exposure group was better than that of the SHS exposure group. No clear seasonal pattern of pulmonary function was found in this study. Aging, SHS exposure, and poor ventilation showed negative effects on most pulmonary function indices. It is recommended to actively publicize the harm of smoking and strengthen house ventilation to improve lung function in local homemakers.

## 1. Introduction

In 2021, approximately 5.521 million people from all over the world suffered from chronic respiratory diseases [1], which work together to gradually deteriorate lung function [2]. The assessment of lung function through pulmonary function tests provides a window into respiratory health, specific pulmonary disorders, and the general heath of individuals [3]. Pulmonary function tests include different kinds of breathing tests which measure how well lung works, which have a long and rich history in the definition, diagnosis, and management of chronic obstructive pulmonary disease (COPD) [4]. Lung function reference values are traditionally based on anthropometric factors, such as weight, height, sex, and age [5].

The pulmonary function indices serve as critical early-warning biomarkers for respiratory disorders. Previous studies on pulmonary function have focused more on male smokers or children with lower immunity [6,7]. The uneven development between rural and urban areas and short healthcare resources have limited the health of pulmonary function of rural people from coming to public attention. Research has long overlooked rural women in Northwest China—a high-risk group enduring chronic indoor pollution. Lung function is influenced by various factors. Associations of the dietary pattern with objective and subjective lung health were found by Feng [8]. Gecili et al. [9] found that localized seasonal variation had an impact on lung function. He et al. [10] found that abnormal pulmonary function in rural residents from Gansu Province, China, was linked to the use of solid fuel and active smoking. This was also proved right by a nationwide representative cohort study in China [11]. Several other studies concluded that pulmonary function was also influenced by other factors such as sex [12], ethnic variations [13], smoking [14], pet ownership [15], outdoor temperature [16], and maternal microplastic exposure [17].

According to the World Health Organization, approximately 2.1 billion people worldwide rely on open fires or inefficient stoves fueled by polluting fuels, generating harmful household air pollution [18]. Women, who predominantly bear the responsibility for cooking, face disproportionately high health risks due to prolonged exposure to cooking smoke, significantly increasing their likelihood of developing noncommunicable diseases, particularly COPD. Supporting this, data from The Lancet indicate that COPD prevalence among women is projected to rise from 7.8% in 2020 to 8.3% in 2050, a relative increase of 6.4%. In contrast, prevalence among men is expected to decline from 13.4% to 10.6% over the same period [19].

Xi’an, a central city in Northwest China, is also known as the starting point of the land-based Silk Road and the 9th National Central City. People from Northwest China share similar habits and customs (e.g., dietary habits, farming practices) influenced by geographical and climatic factors [20]. Rural areas of Xi’an can represent the typical conditions of rural areas of Northwest China. The Fenwei Plain, situated within the vast and populous Northwest China region, serves as one of its representative areas. There is relatively little research focusing specifically on rural women in Northwest China, a population with unique exposure characteristics due to factors like geography, climate and lifestyle. Compared to urban homemakers, rural ones face significantly poorer indoor and outdoor air quality due to substantial air pollutant emissions from solid fuel combustion, thereby adversely affecting regional atmospheric conditions and residents’ health. With greater exposure to diverse air pollution sources and weaker protective awareness, rural homemakers are likely to experience more severe lung function impairment. Therefore, it is necessary for further research in this specific area targeted at female homemakers. The primary objective of this study is to comprehensively investigate the lung function status of homemakers in the Fenwei Plain region and systematically analyze its influencing factors, filling this critical gap in the field. Based on the research findings, evidence-based recommendations will be developed to provide practical guidance for local residents to improve their respiratory health. This study can not only reveal the synergistic mechanisms of multiple factors on lung health, providing a scientific basis for formulating targeted strategies to improving the health of local residents in rural areas, but also fill the gap in lung health research on specific regional female groups, offering evidence-based insights for region-specific respiratory disease prevention strategies.

## 2. Methods

### 2.1. Study Area and Population

The survey was conducted among local homemakers (permanent residents) in three adjacent villages in Lantian County, Xi’an, Shaanxi Province, during winter (4–8 January 2019) and summer (5–9 July 2019). The climate of Lantian County features distinct seasons, with a sharp contrast between summer and winter. Summers are hot and humid with concentrated rainfall, while winters are cold and dry with little rain or snow. Influenced by the monsoons, its key characteristics are hot, rainy summers and cold, dry winters, typifying a continental monsoon climate.

In this study, 72 female homemakers in the surrounding rural areas of Xi’an were recruited to conduct questionnaire surveys and pulmonary function tests in both winter and summer to quantify the lung function of rural homemakers and identify risk factors for pulmonary impairment. This study implemented strict screening for participants. The inclusion criteria were as follows: permanent residents who were female heads of households primarily responsible for winter heating and daily cooking; consistent use of a stable type of solid fuel for over two years; residence, diet, and living conditions similar to the general local population; self-reported good or relatively good health with no history of severe chronic or genetic diseases; all participants were non-smokers and showed no significant respiratory symptoms during the three days preceding the survey; sufficient understanding of the study content and voluntary provision of informed consent; and generally common accommodation, dietary habits, and winter living environment. Exclusion criteria included the following: households with children under the age of 7 (as young children might interfere with the operation of instruments); women who were pregnant or lactating; and individuals unable to cooperate with the survey.

Solid fuel combustion sources are considered a common and persistent local source of specific sources of potential air pollution exposure, and cooking oil fumes and resuspended dust are long-term and continuous exposure sources, as homemakers in this region consistently use solid fuels for cooking (all-year) and heating (winter only). Therefore, when assessing the effects of seasons, only differences in atmospheric pollution and meteorological factors in winter and summer were considered. All 72 participants completed surveys both in winter and summer, 68 of which were included in the analysis, having excluded 4 participants with missing information. This study was approved by the Biomedical Ethics Committee of Health Science Center of Xi’an Jiaotong University (Registration No. 2018-532), and all participants signed informed consent forms.

### 2.2. Pulmonary Function Tests

The purpose of pulmonary function testing is to obtain a comprehensive profile of lung volumes and airflow, including both slow (VC) and forced (FVC) maneuvers. This facilitates the provision of baseline data for subsequent research into the impact of key factors on the lung function of these female homemakers. Trained technicians used a portable spirometer (Pony FX, Cosmed, Rome, Italy) to measure pulmonary function on-site. Each participant was tested at least three times to increase the accuracy. Three measurements were recorded as the valid data when the difference between three measurements was within the error range (5%) of each parameter. Parameters included the following: Vital Capacity (VC), Forced Expiratory Volume in first second (FEV1), Forced Vital Capacity (FVC), Peak Expiratory Flow (PEF), FEV1/VC, FEV1/FVC, Forced Expiratory Flow at 25% of vital capacity (FEF25), Forced Expiratory Flow at 50% of vital capacity (FEF50), and Forced Expiratory Flow at 75% of vital capacity (FEF75). FEV1/FVC is the primary indicator for detecting airflow obstruction. However, its ability to reflect severity is limited as it may appear paradoxically elevated in severe cases due to a significant reduction in FVC. In such scenarios of severe obstruction, FEV1/VC serves as a more reliable alternative ratio to avoid inaccuracies from an incomplete FVC maneuver. Therefore, FEV1/FVC is the standard screening tool, while FEV1/VC is reserved for specific cases where severe obstruction compromises FVC measurement. Both measured values and the percentage of the measured value relative to the predicted values were recorded. Values presented in this study are the average of three valid measurements of each participant.

### 2.3. Data Collection from Questionnaires

Basic information of the participants was collected via questionnaires. The questionnaire was self-designed based on the characteristics of local residents and the actual situations of the villages, not on the basis of existing scales, since there is currently no widely used standardized scale or questionnaire. We have designed and developed our own questionnaire by taking into account various factors and referencing the previous literature used in research studies.

We surveyed and collected 40 parameters in the questionnaire, which were primarily categorized into 3 groups: basic personal information and health status (age, height, weight, BMI, education, disease history, medication history, household income, diet, alcohol consumption, etc.), household environment (presence of a yard, proximity to pollution sources, etc.), and indoor air pollution source and several living habits (ventilation practices, heating energy or fuel type, pet ownership, plant cultivation, incense use, cooking energy or fuel type, etc.). In our study, “clean fuels” refer to clean heating ways by using electricity. This is in contrast to “solid fuels” such as coal and biomass (e.g., wood, crop residues). Ventilation was defined based on the use of exhaust fans during cooking. The classification into “ventilation group” and “non-ventilation group” was determined by whether a fan is chosen by a participant to be used for ≥30 min a day or not during cooking. Second-hand smoke (SHS) exposure was defined based on a “Yes” response to the questionnaire item: “Are you regularly exposed to tobacco smoke from other family members inside your home?” which means that when a family member smokes inside home, it is considered that the participant is exposed to second-hand smoke.

The meteorological data during the sampling periods were recorded and statistically analyzed by Feng et al. [21].

### 2.4. Data Analysis

Questionnaire data were analyzed using Excel 2019 and IBM SPSS Statistics 19.0. A general linear model was used to assess the influence of all kinds of factors on pulmonary function. The effect size of each factor was assessed using Eta squared (η^2^), which represents the proportion of total variance in the dependent variable explained by each independent variable. Based on the η^2^ values, four factors with the highest contribution—age, season, SHS exposure, and kitchen ventilation—were selected for further analysis of their impact on different lung function indicators. Differences between groups were determined using one-way ANOVA or non-parametric tests for normally and non-normally distributed variables, respectively. The differences in pulmonary function indicators between subjects in winter and summer were analyzed using a paired sample t-test. A *p*-value < 0.05 (*) was considered statistically significant.

## 3. Results

### 3.1. Basic Information and General Characteristics of Participants

In this study, the basic information of the subjects is listed in Table 1. The mean age of the valid participants was 61 ± 9 years old (mean ± SD), and the mean value of Body Mass Index (BMI) was 24.9 ± 3.55 kg m^−2^. Approximately 35.3% of the tested subjects had a normal BMI value. In total, 94.1% and 95.6% of the participants used solid fuels in their household in winter and summer, respectively. For heating fuels, most of the participants used coal (36.8%) and biomass (39.7%); only few others chose to use clean fuels (16.2%) and coal mixed with biomass (7.35%). For cooking, most of participants used biomass as energy (88.2% in winter, 94.1% in summer). During cooking, most of the participants did not ventilate (88.2% in winter, 75.0% in summer). About 60% of them were at the risk of SHS exposure both in winter and in summer.

**Figure 1:**
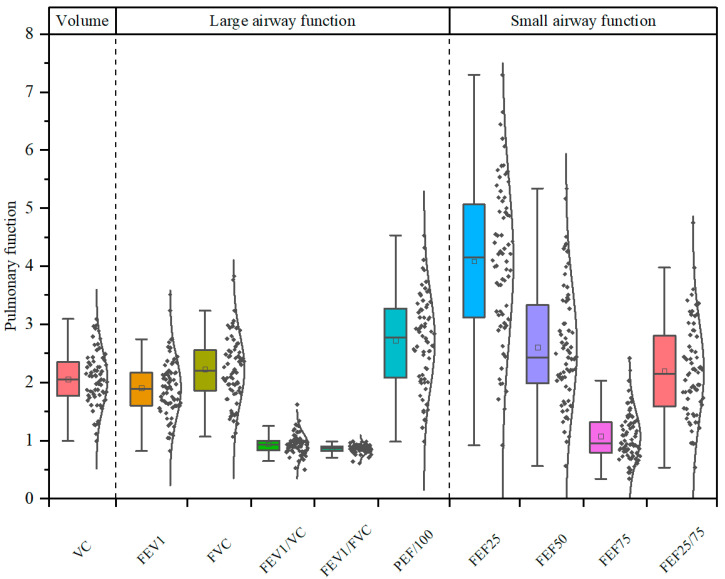
The distribution of main pulmonary function indices for female homemakers (Units: L for VC, FEV1 and FVC; L s^−1^ for FEF25, FEF50, FEF75 and FEF25/75; and L min^−1^ for PEF.).

The data tested for each individual in winter and summer were averaged, and the overall distribution of lung function indices of the subjects was analyzed (Figure 1). The indices measuring lung function can be divided into three categories: lung volume parameters (including VC), large airway function parameters (including FVC, FEV1, FEV1/VC, FEV1/FVC, and PEF), and small airway function parameters (including FEF25, FEF50, FEF75, and FEF25–75). Lung volume parameters can measure the amount of air that can be inhaled and exhaled, and airway function parameters can be used to diagnose abnormal ventilatory function. According to data shown in Figure 1, the average of VC was 2.06 ± 0.48 L. For large airway parameters like FEV1, FVC, FEV1/VC, FEV1/FVC and PEF, the average values were 1.91 ± 0.52 L, 2.23 ± 0.59 L, 0.94 ± 0.18, 0.86 ± 0.07, and 272.37 ± 79.70 L min^−1^, respectively. In contrast, for small airway (FEF25, FEF50, FEF75, FEF25–75) indices, the average values were 4.09 ± 1.35, 2.60 ± 1.02, 1.07 ± 0.42 and 2.20 ± 0.81, respectively. In normal conditions, the values of VC and FVC are approximately equal in healthy individuals. However, in the presence of airflow obstruction, forced expiration can lead to airway closure, resulting in VC being slightly greater than FVC. We believe this discrepancy between FVC and VC in this study may be attributed to external factors such as participant technique during the testing procedure. One possible explanation is suboptimal patient technique during the slow VC maneuver (e.g., an incomplete inhalation or exhalation), which is more common in field studies with older participants who seldom take pulmonary function tests. Additionally, the advanced age of the participants may have contributed to reduced expiratory persistence, which could also have influenced the test results to some extent.

The measured/predicted values of pulmonary function indices were calculated (Table 2). It can be seen that the FEV1/FVC measured/predicted values were less than 1; so, the measured values were lower than the predicted ones, which meant that the indices were at a less healthy level; and that FEF25, FEF50 and FEF75 all had measured values higher than the predicted ones, which meant that the indices were at a healthier level.

### 3.2. The Main Factors Affecting Pulmonary Function

Among the 40 parameters from the questionnaire, 13 parameters were selected for model construction, ultimately yielding the 4 most significantly correlated indicators to lung function discussed in the text, which were age, season, kitchen ventilation during cooking and SHS exposure, while other factors showed low effects (Eta < 1%). The results of between-subject effect tests for season, age, SHS exposure, and kitchen ventilation are shown in Table 3. The variables explained 35.3% of the total variance in all pulmonary function indicators. Age had the largest effect (Eta: 22.3%, range 6.1–84.1%). Indoor ventilation, season, and SHS exposure had comparable effects (Eta: 3.2–5.9%). Age had the largest effects on the FEV1/FVC measured/predicted (55.5%) and FEF50/FEF50 measured/predicted values (84.1%), while kitchen ventilation had the largest effects on the FEF75/FEF75 measured/predicted values (15.0%), seasons had the largest effects on FEV1/VC (15.0%), and SHS exposure had the largest effects on VC (8.9%).

#### 3.2.1. Age

Participants were divided into four age groups based on quartiles: <57 years, 57–63 years, 64–69 years, and >69 years. Statistically significant differences (*p* = 0.000–0.005) were found for all pulmonary function indices except FEV1/VC, FEV1/FVC, and FEV1/FVC Measured/predicted values. The <57-year group showed the highest values, indicating optimal pulmonary function. This study also concluded that significant negative correlations were found between age and pulmonary function indices (except FEV1/VC and FEV1/FVC), confirming the detrimental effect of aging on pulmonary function (see Table 4 for details).

#### 3.2.2. Kitchen Ventilation

Housing in rural Xi’an often consists of single-room dwellings where cooking, sleeping, etc., occur in the same room, lacking separate kitchens. Thus, this study considers that kitchen ventilation reflects overall housing ventilation. Participants ventilating less than 30 min a day were classified as the non-ventilation group, and those ventilating more than 30 min a day as the ventilation group. As shown in Table 5, the mean ages of two groups were similar (61 and 62 years old in the non-ventilation group and the ventilation group, respectively). All pulmonary function indices (except FEV1/FVC and FEV1/FVC Measured/predicted values) of the ventilation group were higher, suggesting that ventilation has a positive impact on pulmonary function. Statistically, there was no significant difference in all indicators between the two groups (*p* = 0.069–0.952).

#### 3.2.3. Seasons

Residents in rural areas of Fenwei Plain, China, have different lifestyles in summer and winter (e.g., heating, cooking, etc.), leading to the air quality indoors being different in summer and winter (usually higher in winter owing to fuel combustion). The ambient concentration of air pollutants is often higher in winter in the Fenwei Plain, China, which also impacts lung function. Previous research was conducted in this area and it was found that the median ambient concentration of PM_2.5_ in winter was more than nine times higher than that in summer [21]. Therefore, the intent of the seasonal comparison was to investigate whether lung function fluctuates in response to pronounced seasonal differences in both outdoor air pollution and indoor heating practices (e.g., solid fuel use for heating occurs only in winter).

Solid fuel usage rates were similar in winter (94.1%) and summer (95.6%) (*p* = 1.000), indicating no seasonal differences in this potential pollution source. Some indices (FEV1, PEF, FEV1/VC, FEV1/FVC, FEF50, FEF75, FEF25–75 and FEF25 Measured/predicted values) were significantly higher in winter, while others (VC, FVC, FEF25, FEV1/FVC and FEF50 Measured/predicted values) were higher in summer. Significant seasonal differences were observed for FEV1/VC, FEV1/FVC, FEF75, and FEF25–75 (Table 6). This indicates a non-significant seasonal pattern of pulmonary function among homemakers.

#### 3.2.4. SHS Exposure

As shown in Table 7, the mean ages were identical (61 years) between the SHS exposed and non-exposed (control) groups (*p* = 0.940). Most pulmonary function indices (except VC, FVC) were higher in the non-exposed group, suggesting a detrimental effect of SHS exposure. Statistically, a significant difference (*p* = 0.034) was found for FEV1/FVC between the two groups.

#### 3.2.5. Summary of Results

Based on the statistical analysis above from 3.2.1 to 3.2.4, the *p*-values for different impacting factors on lung function indicators revealed that age was the most significant factor (*p* < 0.01) for most indicators, with the youngest group (<57 years) showing the best lung function. Seasonal effects were significant (*p* < 0.05) for specific airway indices (e.g., FEV1/FVC, FEF75), with higher values in winter. Second-hand smoke exposure significantly affected only FEV1/FVC (*p* = 0.034), but the non-exposed group had generally better lung function. Overall, kitchen ventilation showed non-significant (*p* < 0.05) yet consistent positive trends. These findings imply the negative impacts of aging, second-hand smoke, and poor ventilation. Upon further in-depth analysis, for example, for the FEF75 measured/predicted value, the average age of the subgroup with values below 1 was 74 years, compared to 59 years for those with values equal to or above 1. This indicates a notable association between age and the FEV1/FVC measured/predicted value, with a tendency for older individuals to exhibit lower values.

## 4. Discussion

### 4.1. Comparison of Pulmonary Function Indices

According to Guidelines for Pulmonary Function Tests of China (2014), normally, FVC/predicted ≥ 80%, FEV1/predicted ≥ 80%, FEV1/FVC/predicted > 92% (or FEV1/FVC > 70%), and two of the three indicators, namely FEF50, FEF75, and FEF25–75, are no less than 65% of the predicted values. The research data for this study all meet the above conditions; that is, the first three values coincide with the guidelines and FEF50 and FEF75 measured/predicted values were more than 65%. This indicates that generally all the participants have healthy lung function based on the above conditions. According to predictive equations and reference values given by Gao et al. [22] about females at age 61–65 years old in Table 8, the differences in all indices between the data in this study and the reference values were all less than 20% and all of them were less than about 10% except PEF; so, the results showed that the homemakers had relatively normal lung function values.

More studies conducted in different areas are also shown in Table 8. A study conducted in Shiyan, Hubei, among 19,128 retired employees of automobile companies, including 10,604 males (41.57%) and 14,907 females (58.43%), provided 25,511 observations and had an average age of 65 years old [23]. They had slightly lower values (except FVC) than the rural homemakers of Xi’an in this study. A total of 3759 adults were recruited from Wenzhou in the study conducted by Tan et al. [24], and the mean age of the study participants was 62.8 ± 10.2 years, with 51.8% female. The results showed that FEV1, FVC, FEF75 were higher than the values observed for the rural homemakers of Xi’an in this study, while PEF, FEV1/FVC, FEF25 were lower. Chen et al. [25] completed a study on evaluating rural-urban differences among patients with Chronic Obstructive Pulmonary Disease (COPD) in Chengdu, China, with a sample of 372 COPD patients from China and found that COPD patients in urban areas had higher FEV1, FVC, and FEV1/FVC values than those in rural areas of Chengdu, China. However, another study carried out by Al-Qerem et al. [26] examined the effect of an urban environment on pulmonary function tests of children by comparing children from an urban (Cairo) and a rural (Shibin El-Kom) area in Egypt and found that living in an urban area decreased the level of FVC, FEV1 and PEF. Meanwhile, Yang et al. [27] enrolled a nationwide sample of adults aged 20 years or older from ten provinces of China among 50,991 participants to represent more diverse geographical regions in China, and it can be concluded that for large airway parameters (FEV1, FVC and PEF), the tested homemakers of Xi’an had lower values. However, for small airway indices, FEF25 and FEF50 were smaller, while the FEF75 and FEF25–75 values were larger in this study than the national average.

### 4.2. Main Pulmonary Function Affecting Factors

Research confirms age as a major COPD risk factor, with older individuals exhibiting poorer pulmonary function [20]. In 2019, the Global Burden of Disease showed that COPD prevalence increased sharply with age from 1990 to 2019, peaking at above 95 years [28]. This study found age had the largest effect size (Eta: 22.3%) on pulmonary function. Significant negative correlations were found between age and all kinds of indices (except FEV1/VC, FEV1, FVC), with the youngest group (<57 years) presenting significantly higher values for large airway (FEV1, FVC, PEF) and small airway (FEF25, FEF50, FEF75, FEF25–75) parameters, which led to a healthier pulmonary function. Although the FEV1/VC and FEV1/FVC values were similar across all groups, the oldest group (>69 years) had the lowest values. Similarly, a study on pulmonary function by Marek et al. [29] among 262 healthy, non-smoking Caucasian males aged 20–90 years demonstrated significant negative correlations between age and all investigated respiratory parameters. A cross-sectional study by Medbø et al. [30] conducted among people over 60 years old in northern Norway revealed that FEV1/FVC significantly decreases with age. These results may be due to age-associated physiological changes: reduced power of lung movement, reduced elastic recoil of lung tissue, increased chest wall rigidity, and narrowing of intervertebral spaces. These changes alone or in combination lead to the degradation of pulmonary function [31].

The effect of ventilation on pulmonary function showed an effect size of 5.9% in this study. Although differences between the ventilation and non-ventilation groups were mostly non-significant, better ventilation correlated with better pulmonary function values (except FEV1/FVC and FEV1/FVC Measured/predicted values) in general. Good ventilation limits indoor pollutant accumulation (especially PM and VOCs from cooking), and therefore reduces humidity and prevents fungal growth, lowering the risk of respiratory disease [32]. Few studies have specifically focused on the impact of ventilation (especially in kitchen) on homemakers’ pulmonary function so far. Only a prospective 9-year cohort study found that improved kitchen ventilation (by using exhaust fans, for example) made a positive and significant difference to pulmonary function [33], which was further supported by the results of this study.

Season and weather influence pulmonary function as well. The results indicate a statistically significant difference in FEV1/FVC in summer and winter, which is influenced by air pollutants’ concentrations and temperature in different seasons. For residents in rural areas of the Fenwei Plain, China, they have different lifestyles in summer and winter (e.g., heating, cooking). The air quality is also different in summer and winter. During this study, winter had significantly higher PM_2.5_, PM_10_, SO_2_, CO, NO_2_ concentrations and lower O_3_ concentrations and temperatures than summer [21], which can make a difference to lung function. Besides FEV1/FVC, significantly higher values for other indices like FEF75, FEF25–75 in winter than in summer might also relate to temperature differences between two seasons. Evidence suggests that an increase in temperature is associated with a decline in pulmonary function, especially in the elderly population. Higher temperatures can cause people (especially the elderly) physiological stress, resulting in respiratory and other systems’ dysfunction [34]. Long-term exposure to O_3_ is independently associated with impaired small airway function [35], as shown in significantly high values of small airway indicators (FEF75, FEF25–75) in summer, which may be related to the inflammation and oxidative stress response induced by human exposure to O_3_ [36]. This study suggests that the FEV1/VC, FEV1/FVC, FEF75, and FEF25–75 values are more sensitive to temperature and O_3_ exposure. Zhang et al. [36] found that O_3_ has a more significant effect on FVC and FEV1, which may indicate the specificity of the impact of different factors on pulmonary function indicators in different studies. No significant seasonal differences were found in other pulmonary function indicators in this study, which is consistent with previous studies [37,38]. The survey conducted by Zhao et al. [38] on pneumonia outpatients and COPD patients in Shenyang suggested that there was no statistical difference in pulmonary function between winter and summer among pneumonia patients.

Smoking is detrimental to health. The 2020 Report on the Health Hazards of Smoking in China [39], issued by the National Health Commission of China, states that smoking causes over 1 million deaths annually, with SHS exposure contributing over 100,000 deaths nationwide. Participants in this study were non-smokers, but 40.4% of them were SHS-exposed, increasing risks for cardiovascular disease, COPD, and upper respiratory illnesses. The results show that most non-SHS-exposed individuals had better large airway indices (except FVC) and all small airway function indices than the SHS-exposed group. Statistically, a significant difference (*p* = 0.034) was found for FEV1/FVC between the two groups. The harmful components in second-hand smoke—reactive oxygen species, nicotine, fine particulate matter, and irritant gases—collectively lead to airflow limitation. This limitation becomes particularly evident during forced exhalation, manifesting spirometrically as a characteristic decrease in the FEV1/FVC ratio. This explains why even without active smoking, individuals with long-term exposure to second-hand smoke can develop lung function impairments analogous to those seen in early-stage COPD. While most differences were not statistically significant, the results indicate that SHS exposure leads to a decline in pulmonary function, particularly affecting small airways. These findings are consistent with domestic and international studies carried out by Chen [40], Du [41] and Flouris [6,42]. The impact of SHS exposure on pulmonary function may be due to the fact that inhaling cigarette smoke activates the vagus nerve, leading to irregular breathing patterns, cough reflex, and bronchial constriction [43]. On the other hand, fibrosis and thickening of the airway wall, especially the subepithelial compartments of small airways, are important components of the pathogenesis of airflow limitation caused by SHS. This study provides new evidence for SHS exposure impacting small airway function.

It should be acknowledged that the relatively small sample size and specific research area may limit the statistical power and the generalizability of the findings. We frame this study as an important initial investigation (or “pilot study”) in this specific region and population, and there is potential for selection bias. We also acknowledge the potential for information bias inherent in the use of self-reported questionnaire data (e.g., ventilation and SHS exposure). We believe this focused approach offers pioneering insights and fills a specific gap in the literature, laying the groundwork for future broader-scale studies to further deepen our understanding of the topic.

## 5. Conclusions

This study examined multiple lung function indicators of 68 female homemakers of rural areas in Lantian, Xi’an, Shaanxi, in winter and summer; all of the indices (except PEF) showed that the homemakers had relatively normal lung function values. That is, all the participants have no serious lung function diseases according to the reference values. However, some factors can still cause impairment of pulmonary function. The cause of pulmonary function impairment is complex. This study demonstrates the negative impact of aging and SHS exposure, the non-significant positive effect of good ventilation, and the differential effects of atmospheric pollutants and meteorological factors (especially O_3_ and temperature) on various pulmonary function indices. Sex, healthcare conditions, regional environment, environmental tolerance, and individual susceptibility may be factors as well [44]. Thus, future research should include diverse populations (e.g., both sexes, COPD patients) to observe the harmful effects of more factors on pulmonary function among different populations. Meanwhile, sample sizes may be increased to enhance the universality of the results. Nevertheless, this study is the first to focus on pulmonary function in Northwest China’s rural residents, providing valuable scientific evidence for risk assessment of pulmonary function and contributing to improving respiratory health among local rural homemakers.

## Figures and Tables

**Table 1:** Basic information of participants.

Information	Winter (N = 68)	Summer (N = 68)
Age (years old)	61 ± 9	
BMI (kg m^−2^)	24.9 ± 3.55	
Underweight (<18.5)	3 (4.41%)	
Normal (18.5–24)	24 (35.3%)	
Overweight (24–28)	27 (39.7%)	
Obese (>28)	14 (20.6%)	
Use of solid fuels		
Yes	64 (94.1%)	65 (95.6%)
No	4 (5.88%)	3 (4.41%)
Heating fuel types		
Coal	25 (36.8%)	/
Biomass	27 (39.7%)	/
Clean fuels	11 (16.2%)	/
Coal mixed with biomass	5 (7.35%)	/
Cooking fuel types		
Coal	2 (2.94%)	1 (1.47%)
Biomass	60 (88.2%)	64 (94.1%)
Clean fuels	6 (8.82%)	3 (4.41%)
SHS exposure		
Yes	28 (41.2%)	27 (39.7%)
No	40 (58.8%)	41 (60.3%)
Ventilation during cooking		
Yes	8 (11.8%)	17 (25.0%)
No	60 (88.2%)	51 (75.0%)

**Table 2:** Measured/predicted values of some pulmonary function indices.

Indices	Average	SD	Maximum	Minimum	Median
FEV1/FVC Measured/predicted values	0.95	0.67	8.73	0.81	0.89
FEF25/FEF25 Measured/predicted values	3.70	0.67	6.36	2.51	3.58
FEF50/FEF50 Measured/predicted values	3.52	0.91	5.29	2.11	3.54
FEF75/FEF75 Measured/predicted values	1.32	0.36	2.29	0.35	1.28

**Table 3:** Effects of influencing factors of pulmonary function index (Eta, %).

Indices	Age	Kitchen Ventilation	Season	SHS Exposure	Total Eta
VC (L)	12.6	11.0	0.3	8.9	32.8
FEV1 (L)	21.1	6.0	4.1	2.6	33.8
FVC (L)	21.9	10.0	0.7	6.7	39.3
PEF (L min^−1^)	12.5	14.4	3.0	0.9	30.8
FEV1/VC	13.6	5.2	15.0	1.4	35.2
FEV1/FVC	6.1	5.6	8.1	5.3	25.1
FEF25 (L s^−1^)	9.3	3.4	0.9	1.7	15.3
FEF50 (L s^−1^)	14.8	0.1	2.1	0.3	17.3
FEF75 (L s^−1^)	12.4	1.4	8.6	2.9	25.3
FEF25–75 (L s^−1^)	14.5	0.0	4.2	0.0	18.7
FEV1/FVC Measured/predicted values	55.5	2.6	1.3	0.7	60.1
FEF25/FEF25 Measured/predicted values	24.7	6.2	0.0	8.4	39.3
FEF50/FEF50 Measured/predicted values	84.1	1.4	0.2	1.3	87.0
FEF75/FEF75 Measured/predicted values	9.4	15.0	6.5	3.1	34.0
Average	22.3	5.9	3.9	3.2	35.3

**Table 4:** Comparison of pulmonary function among homemakers of different ages.

Indices	<57 (N = 36, 26.5%)	57–63 (N = 34, 25%)	64–69 (N = 36, 26.5%)	>69 (N = 30, 22%)	*p*	R^2^
VC (L)	2.36 ^a^	2.01	1.93	1.88	0.000 *	−0.221 *
FEV1 (L)	2.30 ^a^	1.96	1.69	1.62	0.000 *	−0.315 *
FVC (L)	2.65 ^a^	2.33	1.94	1.95	0.000 *	−0.294 *
PEF (L min^−1^)	306.89 ^a^	293.91	248.00	233.53	0.000 *	−0.204 *
FEV1/VC	0.98 ^a^	0.97	0.89	0.87	0.069	−0.091
FEV1/FVC	0.87	0.84	0.88 ^a^	0.84	0.172	−0.105
FEF25 (L s^−1^)	4.69 ^a^	4.26	3.67	3.67	0.005 *	−0.166 *
FEF50 (L s^−1^)	3.10 ^a^	2.69	2.48	2.05	0.001 *	−0.247 *
FEF75 (L s^−1^)	1.34 ^a^	1.00	1.05	0.87	0.000 *	−0.266 *
FEF25–75 (L s^−1^)	2.68 ^a^	2.24	2.03	1.79	0.000 *	−0.254 *
FEV1/FVC Measured/predicted values	0.91	0.89	1.10 ^a^	0.87	0.426	−0.402 *
FEF25/FEF25 Measured/predicted values	4.41 ^a^	3.85	3.33	3.11	0.000 *	−0.318 *
FEF50/FEF50 Measured/predicted values	4.01 ^a^	3.79	3.07	3.17	0.000 *	−0.235 *
FEF75/FEF75 Measured/predicted values	1.76 ^a^	1.39	1.19	0.89	0.000 *	−0.335 *

* Represents statistically significant differences with *p* < 0.05. ^a^ Indicates the highest value of this indicator.

**Table 5:** Comparison of pulmonary function among homemakers with different ventilation conditions.

Indices	Ventilation (N = 111, 81.6%)	Non-Ventilation (N = 25, 18.4%)	*p*
Age (years old)	61 ± 10	63 ± 10	0.406
VC (L)	2.09 ^a^	1.9	0.101
FEV1 (L)	1.94 ^a^	1.75	0.115
FVC (L)	2.27 ^a^	2.03	0.069
PEF (L min^−1^)	275.59 ^a^	255.36	0.290
FEV1/VC	0.94 ^a^	0.94	0.952
FEV1/FVC	0.85	0.87 ^a^	0.544
FEF25 (L s^−1^)	4.12 ^a^	3.94	0.574
FEF50 (L s^−1^)	2.62 ^a^	2.54	0.749
FEF75 (L s^−1^)	1.1 ^a^	0.96	0.194
FEF25–75 (L s^−1^)	2.23 ^a^	2.06	0.378
FEV1/FVC Measured/predicted values	0.89	1.2 ^a^	0.035 *
FEF25/FEF25 Measured/predicted values	3.72 ^a^	3.61	0.492
FEF50/FEF50 Measured/predicted values	3.53 ^a^	3.48	0.806
FEF75/FEF75 Measured/predicted values	1.33 ^a^	1.29	0.595

* Represents statistically significant differences with *p* < 0.05. ^a^ Indicates the highest value of this indicator.

**Table 6:** Comparison of pulmonary function among homemakers in different seasons.

Indices	Winter (N = 68, 50%)	Summer (N = 68, 50%)	*p*
Solid fuel utilization rate	94.1%	95.6%	1.000
VC (L)	2.04	2.07 ^a^	0.681
FEV1 (L)	1.94 ^a^	1.87	0.055
FVC (L)	2.23	2.23 ^a^	0.937
PEF (L min^−1^)	274.96 ^a^	268.79	0.467
FEV1/VC	0.99 ^a^	0.89	0.021 *
FEV1/FVC	0.87 ^a^	0.84	0.003 *
FEF25 (L s^−1^)	4.06	4.12 ^a^	0.613
FEF50 (L s^−1^)	2.65 ^a^	2.55	0.282
FEF75 (L s^−1^)	1.15 ^a^	1.00	0.001 *
FEF25–75 (L s^−1^)	2.27 ^a^	2.13	0.045 *
FEV1/FVC Measured/predicted values	0.89	1.00 ^a^	0.323
FEF25/FEF25 Measured/predicted values	3.72 ^a^	3.68	0.555
FEF50/FEF50 Measured/predicted values	3.49	3.55 ^a^	0.263
FEF75/FEF75 Measured/predicted values	1.32	1.32 ^a^	0.927

* Represents statistically significant differences with *p* < 0.05. ^a^ Indicates the highest value of this indicator.

**Table 7:** Comparison of pulmonary function among homemakers exposed and non-exposed to secondhand smoke.

Indices	SHS Exposure (N = 55, 40.4%)	Non-SHS Exposure (N = 81, 59.6%)	*p*
Age (years old)	61 ± 11	61 ± 10	0.940
VC (L)	2.09 ^a^	2.03	0.563
FEV1 (L)	1.89	1.92 ^a^	0.745
FVC (L)	2.25 ^a^	2.21	0.656
PEF (L min^−1^)	266.78	275.33 ^a^	0.571
FEV1/VC	0.92	0.96 ^a^	0.448
FEV1/FVC	0.84	0.87 ^a^	0.034 *
FEF25 (L s^−1^)	3.96	4.17 ^a^	0.394
FEF50 (L s^−1^)	2.53	2.65 ^a^	0.526
FEF75 (L s^−1^)	1.02	1.11 ^a^	0.257
FEF25–75 (L s^−1^)	2.13	2.25 ^a^	0.456
FEV1/FVC Measured/predicted values	0.89	0.98 ^a^	0.407
FEF25/FEF25 Measured/predicted values	3.67	3.72 ^a^	0.636
FEF50/FEF50 Measured/predicted values	3.51	3.53 ^a^	0.885
FEF75/FEF75 Measured/predicted values	1.32	1.32	0.978

* Represents statistically significant differences with *p* < 0.05. ^a^ Indicates the highest value of this indicator.

**Table 8:** Comparison of pulmonary function indices in different areas.

Indices	Rural Homemakers of Xi’an in This Study	Females at Age 61–65 Years Old [22]	Female of Shiyan * [23]	Adults of Wenzhou [24]	COPD Patients of Urban Chengdu [25]	COPD Patients of Rural Chengdu [25]	Children from Cairo (Urban) * [26]	Children from Shibin El-Kom (Rural) * [26]	Nationwide * [27]
VC (L)	2.06 ± 0.48	2.20 ± 0.50	/	/	/	/	/	/	3.06 ± 0.60
FEV1 (L)	1.91 ± 0.52	1.9 ± 0.4	1.90 ± 0.46	2.0 ± 0.7	1.57 ± 0.58	1.43 ± 0.63	1.69 ± 0.46	1.92 ± 0.43	2.92 ± 0.58
FVC (L)	2.23 ± 0.59	2.1 ± 0.5	2.29 ± 0.56	2.4 ± 0.78	2.49 ± 0.84	2.37 ± 0.86	1.91 ± 0.42	2.10 ± 0.40	2.34 ± 0.52
PEF (L min^−1^)	272.37 ± 79.70	234 ± 66	255.0 ± 90.93	252 ± 108	/	/	201.03 ± 59.51	218.2 ± 68.64	321 ± 83.4
FEV1/VC	0.94 ± 0.18	/	/	/	/	/	/	/	/
FEV1/FVC	0.86 ± 0.07	0.901 ± 0.081	0.84 ± 0.09	0.82 ± 0.13	0.61 ± 0.09	0.58 ± 0.10	0.88 ± 0.09	0.91 ± 0.08	0.80 ± 0.08
FEF25 (L s^−1^)	4.09 ± 1.35	3.7 ± 1.0	/	3.8 ± 1.8	/	/	/	/	4.83 ± 1.35
FEF50 (L s^−1^)	2.60 ± 1.02	2.7 ± 0.8	/	2.6 ± 1.3	/	/	/	/	2.93 ± 1.04
FEF75 (L s^−1^)	1.07 ± 0.42	1.1 ± 0.5	/	1.2 ± 0.75	/	/	/	/	0.97 ± 0.53
FEF25–75 (L s^−1^)	2.20 ± 0.81	2.2 ± 0.8	/	/	/	/	/	/	2.09 ± 0.92

* Represents only female data of the studied subjects which were selected to be compared with this study in Xi’an.

## Data Availability

The raw data supporting the conclusions of this article will be made available by the authors on request.

## Abbreviations

The following abbreviations are used in this manuscript:

COPD	Chronic Obstructive Pulmonary Disease
VC	Vital Capacity
FEV1	Forced Expiratory Volume in First Second
FVC	Forced Vital Capacity
PEF	Peak Expiratory Flow
FEF25	Forced Expiratory Flow at 25% of Vital Capacity
FEF50	Forced Expiratory Flow at 50% of Vital Capacity
FEF75	Forced Expiratory Flow at 75% of Vital Capacity
BMI	Body Mass Index
SHS	Second-hand Smoke
PM	Particulate Matter
PM_2.5_	Particulate Matter with an Aerodynamic Diameter ≤ 2.5 µm
PM_10_	Particulate Matter with an Aerodynamic Diameter ≤ 10 µm
VOCs	Volatile Organic Compounds
